# Myocardial ischemia and previous infarction contribute to left ventricular dyssynchrony in patients with coronary artery disease

**DOI:** 10.1007/s12350-020-02316-9

**Published:** 2020-08-31

**Authors:** Hanna Hämäläinen, Alisa Corovai, Jussi Laitinen, Tiina M. Laitinen, Marja Hedman, Antti Hedman, Antti Kivelä, Tomi P. Laitinen

**Affiliations:** 1 Department of Clinical Physiology and Nuclear Medicine, Kuopio University Hospital, PO BOX 100, Kuopio, 70029 KYS Finland; 2 Institute of Clinical Medicine, University of Eastern Finland, Kuopio, Finland; 3 Department of Cardiothoracic Surgery, Kuopio University Hospital, Kuopio, Finland; 4 Heart Center, Kuopio University Hospital, Kuopio, Finland

**Keywords:** Heart failure, gated SPECT, dyssynchrony, CAD

## Abstract

**Aims:**

The aim of this study was to characterize determinants of left ventricular mechanical dyssynchrony (LVMD) in patients with coronary artery disease (CAD).

**Methods:**

Medical records and results of myocardial perfusion SPECT/CT studies were evaluated in 326 patients with previously diagnosed CAD. LVMD was assessed with the phase analysis of ECG-gated myocardial SPECT. Dyssynchrony was described with phase histogram bandwidth (PHBW), standard deviation (PHSD) or entropy (PHE) values above limit of the highest normal.

**Results:**

Prevalence of LVMD was 29% in CAD patients. Size of the infarction scar and ischemia extent correlated significantly with PHBW, PHSD and PHE (*P *< 0.001 for all). Independent predictors of LVMD were myocardial infarction scar (*P *= 0.004), ischemia extent (*P *= 0.003), and QRS duration (*P *= 0.003). Previous percutaneous coronary intervention and coronary artery bypass grafting did not independently predict dyssynchrony.

**Conclusions:**

Almost one-third of CAD patients had significant LVMD. Dyssynchrony was associated with earlier myocardial infarction and presence of myocardial ischemia. Previous percutaneous coronary intervention and coronary artery bypass grafting did not independently predict dyssynchrony.

**Electronic supplementary material:**

The online version of this article (10.1007/s12350-020-02316-9) contains supplementary material, which is available to authorized users.

## Introduction

Understanding of mechanisms behind development of heart failure progression and therefore, characterization of risk factors predicting left ventricular (LV) dysfunction is important in patients with incipient heart failure. Left ventricular mechanical dyssynchrony (LVMD), asynchronous and dyscoordinate contraction of the LV, has found to be a significant determinant of systolic dysfunction in patients with heart failure.^1^ It limits the LV function and reduces cardiac output.^2^,^3^

Myocardial perfusion imaging (MPI) with ECG-gated single-photon emission computed tomography (SPECT) is widely used to assess possible myocardial ischemia and to study viability of myocardium to optimize treatments of coronary artery disease (CAD) patients.^4^ Phase analysis combined with SPECT MPI is a quite new method and it has become a validated method observing LVMD.^5^,^6^ With phase analysis, it is possible to evaluate automatically the parameters of cardiac synchrony, with high reproducibility, without any discomfort to patient, and regardless of the examiner.^7^ SPECT MPI offers a way to obtain simultaneous evaluation of regional myocardial perfusion, LV function and volumes as well as LVMD.^8^

LVMD has been found to occur even without signs or symptoms of heart disease and it may help to identify patients at higher risk for progression of heart failure.^7^,^8^ The increase in LVMD may be related to multivessel CAD and the severity of coronary artery stenosis.^8^–^10^ LVMD have been seen on the first day after acute myocardial infarction and it has been reported to be present only in the infarcted myocardium or both the infarcted and non-infarcted areas.^11^,^12^ The association between LVMD, myocardial infarct scars and ischemia have earlier been studied with different methods, but there is still versatility between the studies in the determinants of LVMD in patients with CAD.^13^–^16^ Also, invasive cardiac procedures like percutaneous coronary intervention (PCI) or coronary artery bypass grafting (CABG) are important treatments for CAD, but there is still a lack of certainty about how these procedures may contribute to LVMD.

In the present study, the aim was to investigate the relationship between LVMD and myocardial ischemia, myocardial infarction and previous invasive treatments such as PCI and CABG in a population of established CAD.

## Methods

This study was approved by the Research Ethics Committee of the Northern Savo Hospital District.

### Subject Selection/Data Sources

We analyzed medical records of 1191 patients who underwent SPECT/CT examination during January 2009-May 2011 in the Department of Clinical Physiology and Nuclear Medicine, Kuopio University Hospital. Of this population, we included all patients with earlier diagnosis of CAD and with both rest and stress MPI data available to use. Additionally, the 12-lead electrocardiogram (ECG) available from the same MPI examination was the third inclusion criteria. Patients with atrial fibrillation were excluded to avoid bias to LVMD measurements. A total of 326 patients fulfilled all inclusion criteria.

We collected basic characteristics (age, gender, weight, height, body mass index), previous invasive cardiac procedures, such as PCI and CABG, chronic underlying diseases, medications and history of smoking from the medical records. In addition, we analyzed the ECG recordings and MPI data and retrospectively performed the phase analysis. All the information was collected and converted to a single, non-identifiable data set. We verified the whole population as a CAD group and divided the CAD group for subgroups to examine the effects of ischemia or infarct scars. Patients who did not exceed the reference values of phase histogram measurements were defined as non-LVMD subgroup (n = 232) and those who exceeded belonged to LVMD subgroup (n = 94). In addition to that, patients with summed rest score (SRS) points two or more in MPI (described later) were classified into infarct scar subgroup (n = 131) and ischemia% three or more were classified into ischemia subgroup (n = 244). For more precise selection, we studied the effects of location of myocardial infarct scar and the location of myocardial ischemia based on the coronary artery areas to find out, whether there is correlation between increased LVMD and the location of the infarct scar or the ischemic area in myocardium.

Finally, an additional reference group of 52 patients without diagnosed cardiac diseases and normal myocardial perfusion in SPECT imaging was shown in electronic supplementary material in comparison with CAD group. The selection criteria and formation of the reference group is described in details in our previous work.^22^

### Myocardial Perfusion Imaging Protocol

Patients were instructed to avoid caffeine-containing beverages and medications for 24 hours before MPI. Adenosine stress MPI with 99m-Tc-tetrofosmin was performed using 1-day protocol and all patients were studied with both stress and rest phases. At stress phase, adenosine (at rates of 140 μg·kg·min and occasionally due to adenosine-related symptoms at 98 or 70 μg·kg·min) was administered intravenously for 6 minutes with injection of tracer (99m-Tc-tetrofosmin, 300 MBq) at 4 minutes from the onset of adenosine infusion. Low-level exercise was used in combination with adenosine infusion when possible, to avoid influence of extracardial tracer accumulation. Thirty minutes after the tracer injection, ECG-gated SPECT MPI was acquired. For the rest study, patients received an intravenous injection of tracer (99 m-Tc-tetrofosmin, 700 MBq) and were imaged 45 minutes afterwards.

MPI scans were performed in a supine position with a dual detector SPECT/CT system (Philips Precedence; Royal Philips N.V., Amsterdam, Netherlands) with detectors in 90° configurations using 180° body contour orbits with 64 projections and images were acquired with 128 × 128 matrix size. The gated data was acquired with 16 time bins, and with 25 second per angle (patient weight < 100 kg) or 30 second per angle (patient weight > 100 kg). Reconstructions were made retrospectively with HERMES Hybrid Recon Cardiology (Hermes Medical Solution AB, Stockholm, Sweden). Analyses were carried out using automated QPS/QGS2012 software (Cedars-Sinai Medical Center, Los Angeles, California).

### Analysis of LV Perfusion

Stress and rest images were scored according to the 17-segment LV model and a five-point scale (0; normal to 4; absence tracer uptake). The summed rest score (SRS), summed stress score (SSS), and summed difference score (SDS) for perfusion were obtained automatically by software.^17^ SRS from zero to one was considered normal and SRS two or more was considered to represent myocardial scar and SDS two or more was considered to represent myocardial ischemia. We defined the location of an infarct by using SRS point together with coronary arterial areas as described in EANM/ESC procedural guidelines for MPI.^4^ We added the perfusion points in each of these 3 arterial areas (in left anterior descendens [LAD] there were 7 segments, in left circumflex [LCX] 5 and in right coronary artery [RCA] 5 segments) to find out the location of an infarct or ischemia. If there were 2 or more SRS points in LAD area and not in other areas, this was classified as LAD infarct subgroup (LCX and RCA infarct subgroups were determined accordingly). If there were 2 or more SRS points which were divided over the myocardium and not in a specific arterial area, or if there were 2 or more SRS points in two or three of the arterial areas, the case was classified into mixed infarct group.

Furthermore, co-registration of rest and stress images and voxel-by-voxel estimation of differences described by Slomka et al. was also used in quantification of ischemia percentage.^18^ Perfusion defect area relative to total LV area (Extent%) was also shown.^19^

### Quantification of LV Size, Systolic Function and Mechanical Dyssynchrony

In the QGS algorithm, the mid-myocardial LV surface was first computed and endo- and epicardial surfaces were computed subsequently. The end-diastolic volume (EDV), end-systolic volume (ESV), stroke volume (SV) and ejection fraction (EF) were calculated from the volumes determined by the endocardial surface in different time frames.

LVMD was assessed from the ECG-gated SPECT with the phase analysis method. Phase analysis detects regional count changes in 16 time bins to observe the variation in LV wall thickness throughout the cardiac cycle. This is done for 1008 spatial points covering the whole LV area. The onset of mechanical contraction is assessed for each spatial points and the information is presented as a phase histogram which describes distribution of the timing of LV regional onset of mechanical contraction as a function of the length of the R-R-interval.^20^ The phase histogram reflects the heterogeneity of regional LV contraction in timing: the greater the PHBW, PHSD, and PHE are, the more dyssynchronic the contraction is.^21^

The definition of having LVMD was the values of PHBW, PHSD and/or PHE above the limit of the highest normal based on our reference material (PHBW > 63.7°; PHSD > 26.5°; PHE > 63.7%).^22^

### Statistical Analysis

One-sample Kolmogorov–Smirnov test was used to test the normality of distribution for continuous variables. Descriptive statistics is presented as mean ± standard deviation. In the case of continuous variables *T* test was used to test the statistical significance of difference between two independent samples. One-way ANOVA with least significant difference post hoc algorithm was used to test the differences between the mean values of the more than two groups. For categorical variables the Chi square test or Fisher´s exact test, when the expected value in each cell was less than 5, were used. Correlations were tested using Pearson´s bivariate correlations.

Logistic regression analysis was performed to find out the predictors of LVMD. At first, the logistic regression model included age, sex and variables which differed between non-LVMD and LVMD subgroups and therefore were possible predictors of LVMD.

Tests were two-sided and significance was present if *P *< 0.05. All statistical calculations were performed with the SPSS for Windows program (SPSS 24.0, Chicago, IL).

## Results

### Baseline Characteristics

The characteristics of overall patient population are summarized in Table 1. CAD group included all patients with previously diagnosed CAD, and many of them also had previous myocardial infarction and previous invasive cardiac procedures like PCI (n = 127; 39%) and CABG (n = 111; 34%). Patients with LVMD had more myocardial infarct scars (*P *< 0.001) and they were more frequently males compared to those without LVMD (*P *= 0.048). PCI had been done for 39% of the patients in both LVMD and non-LVMD group (*P *= 0.924).

**Table 1:** Clinical characteristics of the study population [Mean ± SD/n (%)]

	CAD group (n = 326)	Non-LVMD (n = 232)	LVMD (n = 94)
Backround information			
Age (years)	68 ± 10	68 ± 10	69 ± 11 NS
Male	198 (61%)	133 (57%)	65 (69%)*
Weight (kg)	83 ± 18	82 ± 17	84 ± 19 NS
Height (cm)	167 ± 10	166 ± 9	169 ± 10**
BMI (kg·m²)	29.5 ± 5.4	29.6 ± 5	29.2 ± 5 NS
Systolic BP	145 ± 23	148 ± 23	140 ± 23***
Diastolic BP	74 ± 10	74 ± 11	75 ± 9 NS
Medication			
Betablocker	286 (88%)	205 (88%)	81(86%) NS
ACE/AT	231 (71%)	164 (71%)	67 (71%) NS
Calcium blocker	104 (32%)	82 (35%)	22 (23%) *
Statin	273 (84%)	195 (84%)	78 (83%) NS
ASA	243 (75%)	176 (76%)	67 (71%) NS
Diabetes medication	88 (27%)	64 (28%)	24 (26%) NS
Anticoagulant	134 (41%)	84 (36%)	50 (53%) *
Nitrate	*202 (62%)*	*146 (63%)*	56 (60%) NS
Diuretic	*118 (36%)*	*81 (35%)*	37 (39%) NS
Antiarrythmic	*4 (1%)*	*2 (1%)*	2 (2%) NS
Comorbidities			
Diabetes	106 (33%)	75 (32%)	31 (33%) NS
Hypertension	250 (77%)	185 (80%)	65 (69%) NS
Kidney disease	22 (7%)	12 (5%)	10 (9%) NS
Pulmonary disease	90 (28%)	64 (28%)	26 (28%) NS
Cancer	37 (11%)	25 (11%)	12 (13%) NS
Myocardial infarct scar (SRS ≥ 2)	131 (40%)	72 (31%)	59 (63%) ***
PCI	127 (39%)	90 (39%)	37 (39%) NS
CABG	111 (34%)	71 (31%)	40 (43%) *
LBBB	23 (7%)	7 (3%)	16 (17%)

Statistical significance between non-LVMD and LVMD groups: *P < 0.05, **P < 0.01, ***P < 0.001

### Incidence of LV Dyssynchrony

About one-third (29%) of all CAD patients had LVMD. The prevalence of LVMD raised to 45% in subpopulation with infarct scar in MPI. In the subgroup of patients with earlier PCI (n = 127), the prevalence of LVMD was 29% and in patients with earlier CABG (n = 111) the prevalence of LVMD was 36%.

Table 2 reports the results of myocardial perfusion scores, LV volume parameters and ECG results in the all CAD patients and in non-LVMD and LVMD groups. The perfusion scores and LV volumes were statistically significantly higher and EF was lower in the LVMD group compared to non-LVMD group. SDS was comparable between the two groups whereas SRS, SSS, ischemia% and extent% were higher in LVMD group as compared with non-LVMD group. Furthermore, QRS duration was significantly longer in LVMD group as compared to non-LVMD group.

**Table 2:** Perfusion scores, volume parameters and ECG recordings in the CAD, left ventricular dyssynchrony and non-dyssynchrony groups (mean ± SD)

Parameter	CAD	Non-LVMD	LVMD
	n = 326	(n = 232)	(n = 94)
Perfusion scores			
Ischemia%	4.2 ± 2.6	3.7 ± 2.4	5.2 ± 2.9***
Extent%	6.5 ± 9.3	4.2 ± 6.7	12.1 ± 12.1***
SDS	2.0 ± 2.2	1.9 ± 2.2	2.3 ± 2.3 NS
SRS	3.2 ± 5.0	1.9 ± 3.4	6.3 ± 6.6***
SSS	5.4 ± 5.8	4.0 ± 4.7	8.8 ± 6.8***
Volume parameters			
EDV (ml)	111.8 ± 60.9	97 ± 47	149 ± 74***
ESV (ml)	50.3 ± 50.4	35 ± 31	89 ± 66***
SV (ml)	61.6 ± 18.8	62 ± 19	60 ± 19 NS
EF (%)	61.5 ± 16.4	68 ± 12	46 ± 17***
ECG recordings			
Heart rate at rest (bpm)	95.0 ± 23	95 ± 22	94 ± 24 NS
QRS (ms)	107 ± 25	101 ± 21	121 ± 29***
PQ (ms)	185 ± 32	182 ± 30	191 ± 38*
QT (ms)	398 ± 36	398 ± 32	400 ± 44 NS
RBBB	23(7%)	16(7%)	7(7%) NS
LBBB	23(7%)	7(3%)	16(17%)***

SDS, Summed difference score; SRS,  Summed rest score; SSS,  Summed stress score; EDV,   End diastolic volume; ESV,  End systolic volume; SV, Stroke volume; EF,  Ejection fraction

Statistical significance between non-LVMD and LVMD groups: *P < 0.05, **P < 0.01, ***P < 0.001

Patients in CAD group had statistically significantly increased amount of ischemia, larger infarction scars and LVMD as well as decreased volume parameters compared to reference group (more detailed data are shown in the electronic supplementary material).

Table 3 presents the phase analysis results both in the rest and stress studies in different subgroups of the patients. In the ischemia group, all phase analysis parameters were higher than in non-ischemia group (*P *< 0.05). The same was detected between the infarct and non-infarct groups. LVMD was significantly increased in stress compared to rest study in the whole CAD group (*P *< 0.001), as well as other subgroups except for in the LVMD group (NS). The change in phase analysis measurements (ΔPHBW, ΔPHSD and ΔPHE) between the rest and stress studies was not statistically significant between the non-infarct and infarct patients (*P *> 0.05) or between the non-ischemic or ischemic patients (*P *> 0.05 for all). In LVMD group, this change seemed to be smaller than in non-LVMD patients (*P *< 0.005 for all).

**Table 3:** Phase histogram parameters in rest and stress studies in the CAD group and subgroups (mean ± SD)

	CAD	Non-LVMD	LVMD	Non-Infarct	Infarct	Non-ischemic	Ischemic
	(n = 326)	(n = 232)	(n = 94)	(n = 195)	(n = 131)	(n = 82)	(n = 244)
PHBW rest	48.5 ± 27	36.2 ± 10	78.8 ± 31 ***	41.5 ± 18	58.9 ± 34 ***	42.7 ± 28	50.4 ± 27 **
PHBW stress	56.7 ± 29^###^	48.5 ± 21^###^	76.9 ± 34 NS***	48.5 ± 20^###^	68.8 ± 35^###^ ***	49.7 ± 27 ###	59.0 ± 29 ^###^ **
PHSD rest	14.1 ± 8	10.8 ± 4	22.2 ± 8***	12.3 ± 6	16.7 ± 9 ***	12.7 ± 8	14.5 ± 7
PHSD stress	16.0 ± 8 ^###^	13.8 ± 6 ^###^	21.3 ± 9 NS***	14.0 ± 6 ^###^	18.9 ± 10 ^##^ ***	14.5 ± 8 ^##^	16.5 ± 8 ^###^ *
PHE rest	59.8 ± 6	57.1 ± 4	66.7 ± 4***	58.2 ± 5	62.3 ± 6 ***	57.9 ± 6	60.5 ± 6 ***
PHE stress	62.8 ± 6 ^###^	61.0 ± 5 ^###^	67.3 ± 5 NS***	61.2 ± 5 ^###^	65.3 ± 6 ^###^ ***	60.9 ± 6 ###	63.5 ± 6 ^###^ ***

Statistical significance between non-LVMD and LVMD group, infarct and non-infarct group and ischemic and non-ischemic group are shown as *P < 0.05, **P < 0.01, ***P < 0.001 and statistical significance between rest and stress studies in each group as ^#^*P *< 0.05, ^##^*P *< 0.01, ^###^*P *< 0.001. In non-infarct group, SRS < 2 and infarct group SRS ≥ 2, non-ischemic group ischemia% < 3 and in ischemic group ischemia%  ≥ 3

### Infarct scar, Ischemia and LV Dyssynchrony

**Figure 1:**
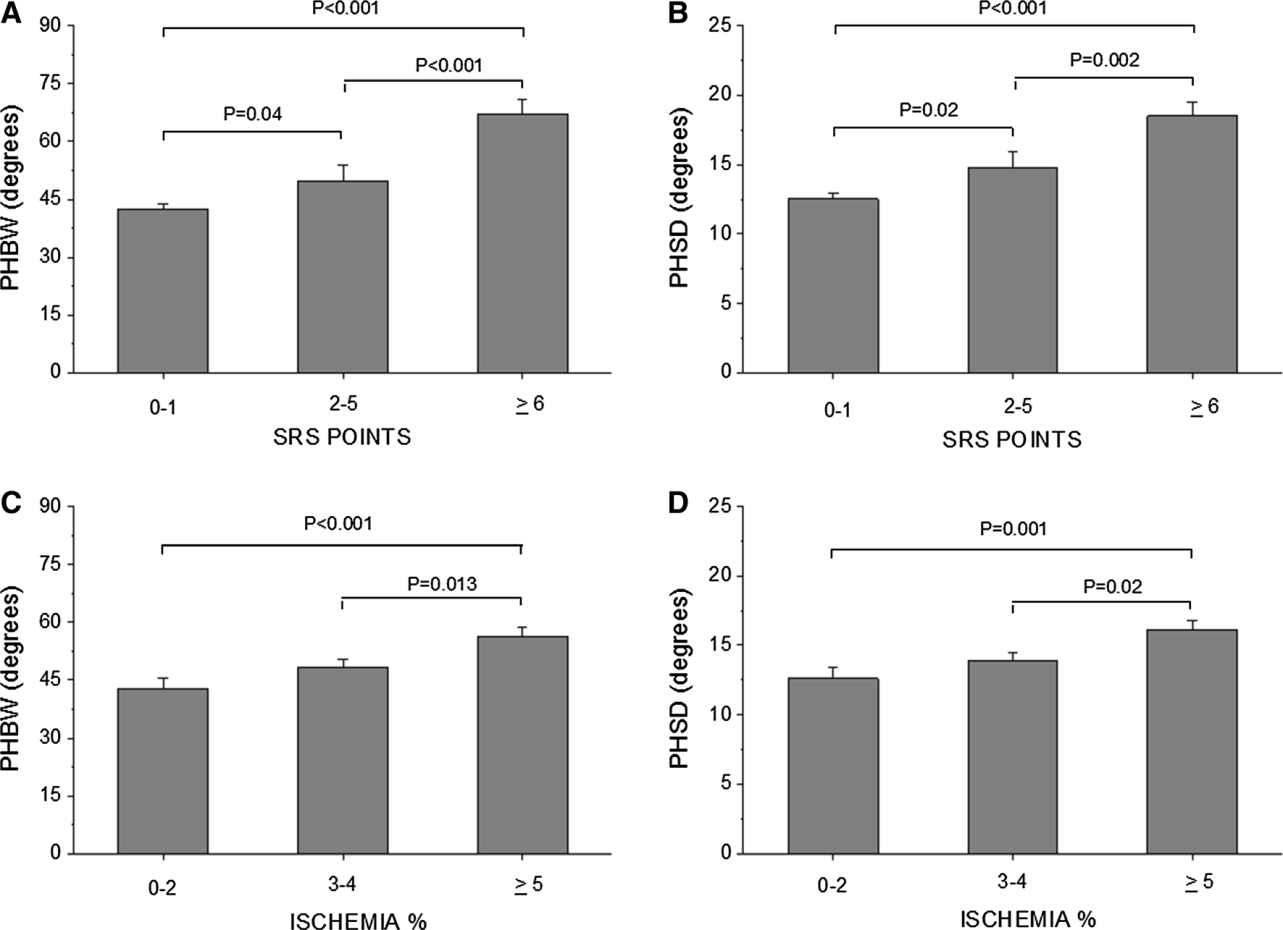
Relationship between **A** infarct scar (SRS points) and phase histogram bandwidth, **B** infarct scar and phase histogram standard deviation, **C** ischemia % and phase histogram bandwidth and **D** ischemia % and phase histogram standard deviation

**Figure 2:**
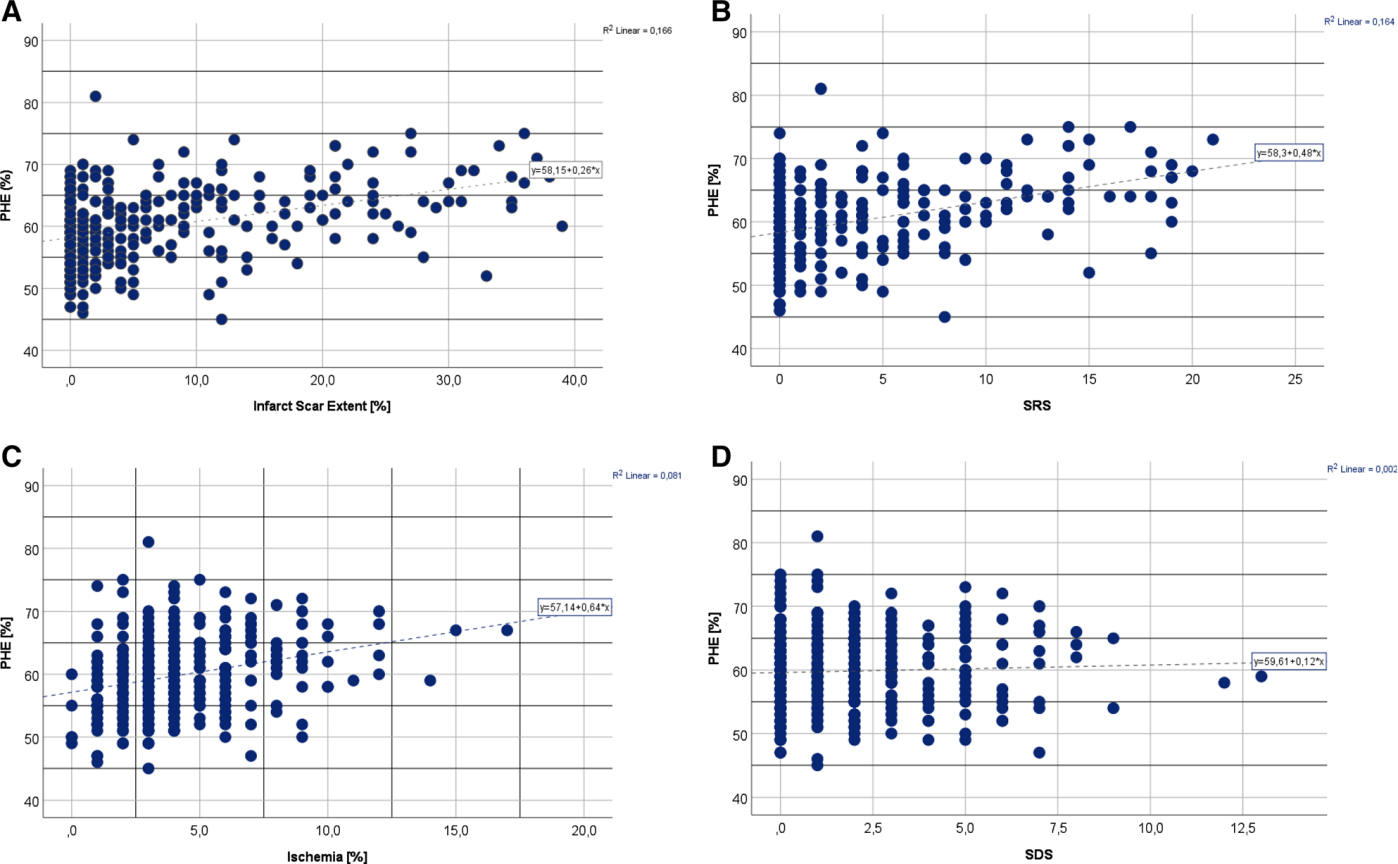
Correlation between phase histogram entropy (PHE) and infarction scar (**A** shows infarction scar as Extent% and **B** as SRS points) and myocardial ischemia (**C** as ischemia% and **D** as SDS points)

Figure 1 shows the relationship between infarct scar, ischemia percentage and phase histogram measurements. The correlations between the phase histogram parameters and parameters reflecting myocardial ischemia and infarct in the whole CAD population are shown in Table 4 and as scatter plot in Figure 2. Phase histogram parameters correlated statistically significantly with values reflecting infarct scar and ischemia% of myocardium, whereas SDS was not statistically significantly correlated with phase analysis measurements.

**Table 4:** Pearson correlation coefficients in the CAD group (n = 326)

	Histogram BW	Histogram SD
Extent (%)	0.429***	0.377***
SRS	0.433***	0.380***
LAD SRS	0.317**	0. 260***
LCX SRS	0.230***	0.214***
RCA SRS	0.263***	0.239***
Ischemia (%)	0.159**	0.159**
SDS	− 0.018 NS	− 0.012 NS

Extent, Infarct extent (%); SRS, summed rest score; LAD, left anterior descendens; LCX, left circumflex; RCA,  Right coronary artery; Ischemia, Myocardial ischemia; SDS,  Summed rest score

Statistical significance *P < 0.05, **P < 0.01, ***P < 0.001

In the subanalysis, there were 33 patients with an infarct scar only in LAD area, 21 patients with LCX areal infarct, 28 patients with RCA areal infarct and 37 patients with mixed areas infarcts. PHBW was highest in the mixed infarct group (68.3 ± 36°) and second highest in the LAD infarct group (58.8 ± 33°) and smallest in the LCX infarct group (52.5 ± 28°). However, phase histogram measurements did not differ statistically significantly between the groups. Chi Square test showed no statistically significant difference in the prevalence of LVMD between these four infarct groups (*P *= 0.054). After excluding the mixed group from the analysis, there were not statistically significant difference in the prevalence of LVMD between LAD, LCX or RCA areal infarct scar (*P *= 0.442 NS). To elaborate the effects of vessel distribution of infarction more precisely, the regression analysis showed that if infarction (described by SRS points) was located in only 1 vessel distribution, it had less effect to increased LVMD (*B *= 0.894, *P *= 0.005) compared to 2 or more vessel distribution location (*B *= 1.736, *P *< 0.001). Moreover, there were no statistically significant differences in phase analysis measurements in patients with detected ischemia in different arterial areas based on SDS points.

### Logistic Regression Analysis

The logistic regression model took into consideration age and sex as fundamental information and as potential predictors of LVMD a set of variables thought to characterize different features of CAD (ischemia extent, infarction scar extent, CABG). Remodeling related to CAD was characterized by EDV and electrical dyssynchrony by QRS duration. According to the analysis, larger infarction extent%, larger ischemia%, and longer QRS duration independently predicted LVMD (Table 5). Previous CABG did not statistically significantly predict dyssynchrony. In analysis standardized with age, sex, myocardial ischemia and previous myocardial infarction, PCI or CABG did not influence on the amount of LVMD.

**Table 5:** Logistic Regression predicting left ventricular mechanical dyssynchrony

	*B*	S.E.	Wald	*P*	OR
Sex	0.405	0.36	1.265	0.261	0.667
Age	0.001	0.016	0.009	0.925	0.999
CABG	0.332	0.305	1.184	0.277	1.394
LBBB	0.907	0.581	2.431	0.119	2.476
Infarct(extent)	0.054	0.019	8.403	0.004**	1.056
Ischemia%	0.168	0.057	8.618	0.003**	1.183
EDV(ml)	0.007	0.004	3.649	0.056	1.007
QRS (s)	20.642	6.853	9.073	0.003**	921639861

Statistical significance; *P < 0.05, **P < 0.01, ***P < 0.001

In additional logistic regression analysis in which EF was also added into the model, significant predictors of LVMD were male sex (*B *= 1.11, *P *= 0.015), ischemia (*B *= 0.181, *P *= 0.005), larger EDV (*B *= − 0.011, *P *= 0.005), low EF (*B *= − 0.154, *P *< 0.001) and longer QRS duration (*B *= 30.1, *P *< 0.001), but not infarct scar extent (*B *= − 0.006, *P *= 0.787).

## Discussion

In this study, the prevalence of LVMD was 29% among CAD patients. LVMD was associated with earlier myocardial infarction and presence of myocardial ischemia. The larger the infarct scar was, the more advanced LVMD was observed. Previous PCI and CABG did not independently predict LVMD. In CAD patients LVMD became even more evident in stress compared to rest study.

Observed LVMD prevalence in this study is about as high as in the earlier study, in which the prevalence among CAD patients was 38%; it was more prevalent in patients with multivessel disease and associated with more impaired measures of LV perfusion and depressed LV systolic function but finally, it could only be predicted by ischemic burden and larger LV.^8^ In our study also, the patients with mixed infarct in MPI, had the highest LVMD values, which might indicate that patients with multivessel disease, are more susceptible for LVMD.

Another study detected that the overall prevalence of LVMD was 6.5% but it reached even 42% in the presence of certain risk factors (male sex and QRS > 120 ms).^7^ They also found a significant correlation between history of CAD and LVMD; the risk for LVMD was about 5 times higher in CAD patients than those without CAD (prevalence ratio 4.44), and the previous myocardial infarct was strongly associated with LVMD, which is in line with our findings. Our study showed that the prevalence of dyssynchrony was highest among patients with previous myocardial infarct and in patients with visible infarct scars in MPI. Earlier studies have reported that LVMD was especially advanced in patients with old myocardial infarction and the extent of myocardial perfusion defect was an independent predictor for LVMD.^23^,^24^ In our study, myocardial perfusion defect measured with extent%, was one of the independent predictors of LVMD. Another study used 3-dimensional echocardiography to quantify LVMD in CAD patients without regional wall motion abnormality and found out that CAD patients still demonstrated increased LVMD compared with healthy controls.^10^

Logistic regression analysis of this study showed that significant predictors of LVMD were myocardial ischemia, infarction scar extent and QRS duration. In additional analysis, when EF was included in the model, significant predictors of LVMD were male sex, ischemia, larger end-diastolic volume, low ejection fraction and longer QRS duration, but not infarct scar extent. The latter model indicates how well the entire bundle of predictors predicts LVMD but it does not indicate that infarct scar would be redundant information. When including EF into the model, the analysis meets the problem of multicollinearity; patients with infarction scar have often decreased EF.^19^ Therefore, after adjustment of EF, the association between infarct scar and LVMD may become limited.

It has been acknowledged, that the greater the amount of LVMD was, the more perfusion defects and infarct scars were present, and myocardial infarction size was an independent predictor of LVMD.^14^,^24^ An area in which the myocardial infarction locates, has also thought to matter to the amount of LVMD. The extent and severity of perfusion and regional wall motion abnormalities have found to be most prominent in the myocardial region supplied by LAD coronary artery, which may explain significantly worse post-stress LV function after anterior acute myocardial infarction.^25^ We found statistically significant correlation between SRS as well as extent% and increased amount of dyssynchrony. In our study, LAD infarct tended to be associated with higher dyssynchrony values compared to LCX or RCA infarct, but the difference was not statistically significant. One reason for that might be the low number of patients in each of those subgroups. Additionally, logistic regression analysis showed that if infarction scar located in 2 or more vessel territories compared to 1 vessel distribution, it had more value to increased LVMD.

In addition to previous myocardial infarction, the correlation between LVMD parameters and ischemia% was statistically significant whereas correlation between SDS and LVMD was not. That suggests that ischemia% might be more sensitive parameter to describe the functional significance of ischemia in the myocardium than SDS. One reason for that might be the fact that voxel-by-voxel estimation technique (Slomka et al.) compared to traditional technique has shown better ability to detect borderline-abnormal findings in patients who had coronary stenosis confirmed by coronary angiography.^17^,^18^ Therefore, it is not unexpected that we found slight, but statistically significant difference in ischemia extent but not in SDS when comparing LVMD and non-LVMD groups. LVMD has found to be associated with inducible ischemia in patients with chest pain and normal coronary arteries which supports our findings in a more clinical view.^15^

In multivessel CAD, more advanced LVMD have been detected at stress compared to rest, as in our study.^9^,^26^ Some studies have shown that stress-induced increasing of LVMD is observed only in patients with perfusion abnormalities, others have stated that the correlation of LVMD with reversible perfusion defects was not significant and some have showed that stress-induced ischemia increases the degree of intraventricular dyssynchrony, as discussed in our previous study.^7^,^15^,^22^,^26^,^27^ We found that in the pooled population and in subgroups of CAD patients with infarct, ischemia and normal LV synchrony at rest, BW, SD and entropy were systematically higher at stress compared to rest. This observation may be explained by the influence of ischemia burden on LV synchronicity or overestimation of LVMD due to inferior image quality related to lower injected activity in stress imaging. However, in those patients, who showed LV dyssynchrony already at rest, BW, SD and entropy were comparable between rest and stress. We speculate that if LVMD is present already at rest further influence of stress on mechanical synchrony becomes limited.

In the present study, earlier PCI did not independently predict dyssynchrony. “Earlier PCI” in this study meant that according to medical history, PCI had been done before SPECT, but the precise timeline between PCI and SPECT was not known. Although this study did not find statistically significant association between earlier PCI and LVMD in long term, this does not prove that that PCI does not contribute to LV synchronicity. Earlier, even the protective value of PCI to LVMD is supported by the studies in which the most of are focused on examining the acute effects of PCI and have been done mostly with tissue Doppler echocardiography.^28^,^29^ Elective PCI as well as PCI in acute phase ST elevation infarct has found to improve LVMD significantly.^28^,^29^ LVMD within 48 hours after PCI has seemed to predict increase in LV size and deterioration of LV systolic function after 6 months follow up.^30^ This suggests that a significant degree of LVMD is predictive of adverse LV remodeling and may offer a possibility to identify patients at risk for LV remodeling early after infarction and to consequently emphasize intensive treatments for these patients.

In addition to PCI, it is commonly known, that CABG surgery induces paradoxical septal wall motion; about 60.5% of patients who had undergone CABG, had paradoxical septal motion quantified by SPECT.^31^ Use of SPECT MPI offers a way to study whether paradoxical movement is accompanied by ischemia or infarction.^31^ It has also been suggested, that abnormal septal motion might not be caused by ischemic insult but might occur due to an increase in anterior cardiac mobility after incision of pericardium.^32^ It seems that entire LV translocates anteriorly in systole after CABG, and evidence of septal infarction was not present on MRI to explain such paradoxical movement.^33^ Despite of this significant abnormal movement, the history of prior CABG does not seem to affect or impact LVMD indices.^34^ This is in line with our observations; previous CABG did not contribute to the values of dyssynchrony independently. LVMD seemed to be slightly increased in CABG patients compared to non-CABG patients in our extra-analysis, but this study setting could not examine the direct effects of CABG to LVMD which could be done by comparing results before and after CABG. Postoperative LVMD may reflect late reverse remodeling and potential of further functional improvement in patients with patent grafts and preserved perfusion reserve after CABG.^35^

To conclude, LVMD seems to be in close relation with CAD, myocardial infarct and ischemia. This connection expresses the importance of CAD in the pathophysiology of heart failure. The adequate prevention and treatment of CAD might prevent LVMD and thus decrease the risk for upcoming heart failure.

### Limitations

The most important limitation was the retrospective nature of the study. All patients were sent to SPECT MPI for medical reasons and CAD patient selection was made based on the previous medical records and written diagnosis of CAD. This made the CAD patient group a versatile group, which might have introduced some bias in the patient selection. However, the present data represent a picture of the actual population of subjects submitted to MPI.

## Conclusions

A considerable part of CAD patients had significant LVMD. In this study, CABG or PCI did not independently predict dyssynchrony, but previous myocardial infarct, myocardial ischemia as well as large EDV did.

## New Knowledge Gained

LVMD is common in CAD patients and is associated with presence of both reversible and irreversible perfusion defects. This connection highlights the importance of detecting and treating of CAD to prevent possible upcoming heart failure.

## Electronic supplementary material

Below is the link to the electronic supplementary material.

Electronic supplementary material 1 (PPTX 248 kb)

## Abbreviations

CAD: Coronary artery disease
LVMD: Left ventricular mechanical dyssynchrony
PHBW: Phase histogram bandwidth
PHSD: Phase histogram standard deviation
PHE: Phase histogram entropy
PCI: Percutaneous coronary intervention
CABG: Coronary artery bypass graft
LV: Left ventricle
EF: Ejection fraction
SPECT: Single-photon emission computed tomography
