# Supplementary material for: Myocardial ischemia and previous infarction contribute to left ventricular dyssynchrony in patients with coronary artery disease
Source: J Nucl Cardiol. 2020 Aug 31;28(6):3010–20. doi: 10.1007/s12350-020-02316-9 (PMC8709814; doi:10.1007/s12350-020-02316-9)
Supplement: Supplementary file 1 — Electronic supplementary material 1 (PPTX 248 kb) [file 12350_2020_2316_MOESM1_ESM.pptx]

## Slide 1
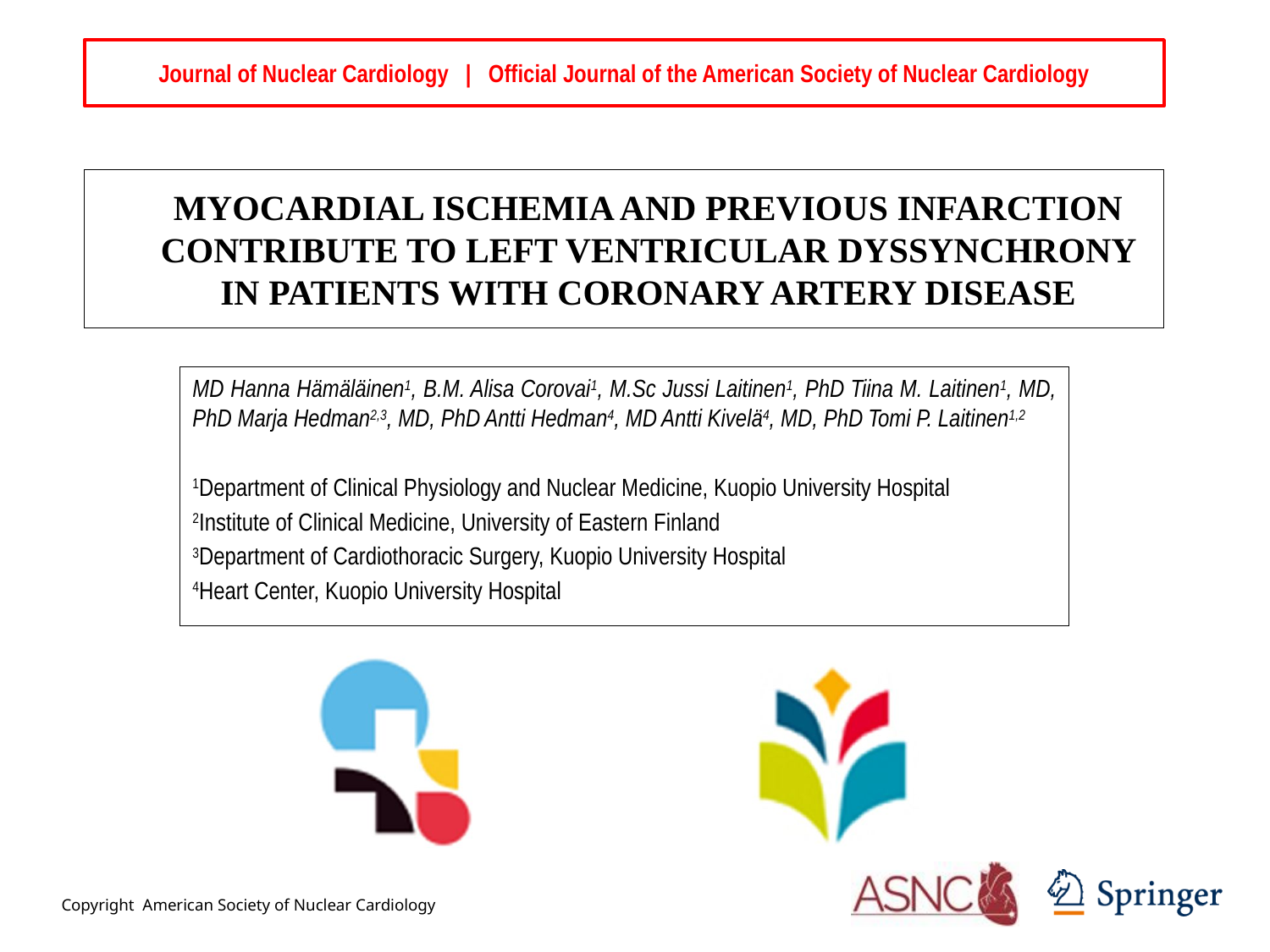

Journal of Nuclear Cardiology | Official Journal of the American Society of Nuclear Cardiology
# MYOCARDIAL ISCHEMIA AND PREVIOUS INFARCTION CONTRIBUTE TO LEFT VENTRICULAR DYSSYNCHRONY IN PATIENTS WITH CORONARY ARTERY DISEASE
MD Hanna Hämäläinen1, B.M. Alisa Corovai1, M.Sc Jussi Laitinen1, PhD Tiina M. Laitinen1, MD, PhD Marja Hedman2,3, MD, PhD Antti Hedman4, MD Antti Kivelä4, MD, PhD Tomi P. Laitinen1,2
1Department of Clinical Physiology and Nuclear Medicine, Kuopio University Hospital
2Institute of Clinical Medicine, University of Eastern Finland
3Department of Cardiothoracic Surgery, Kuopio University Hospital
4Heart Center, Kuopio University Hospital
Copyright American Society of Nuclear Cardiology

## Slide 2
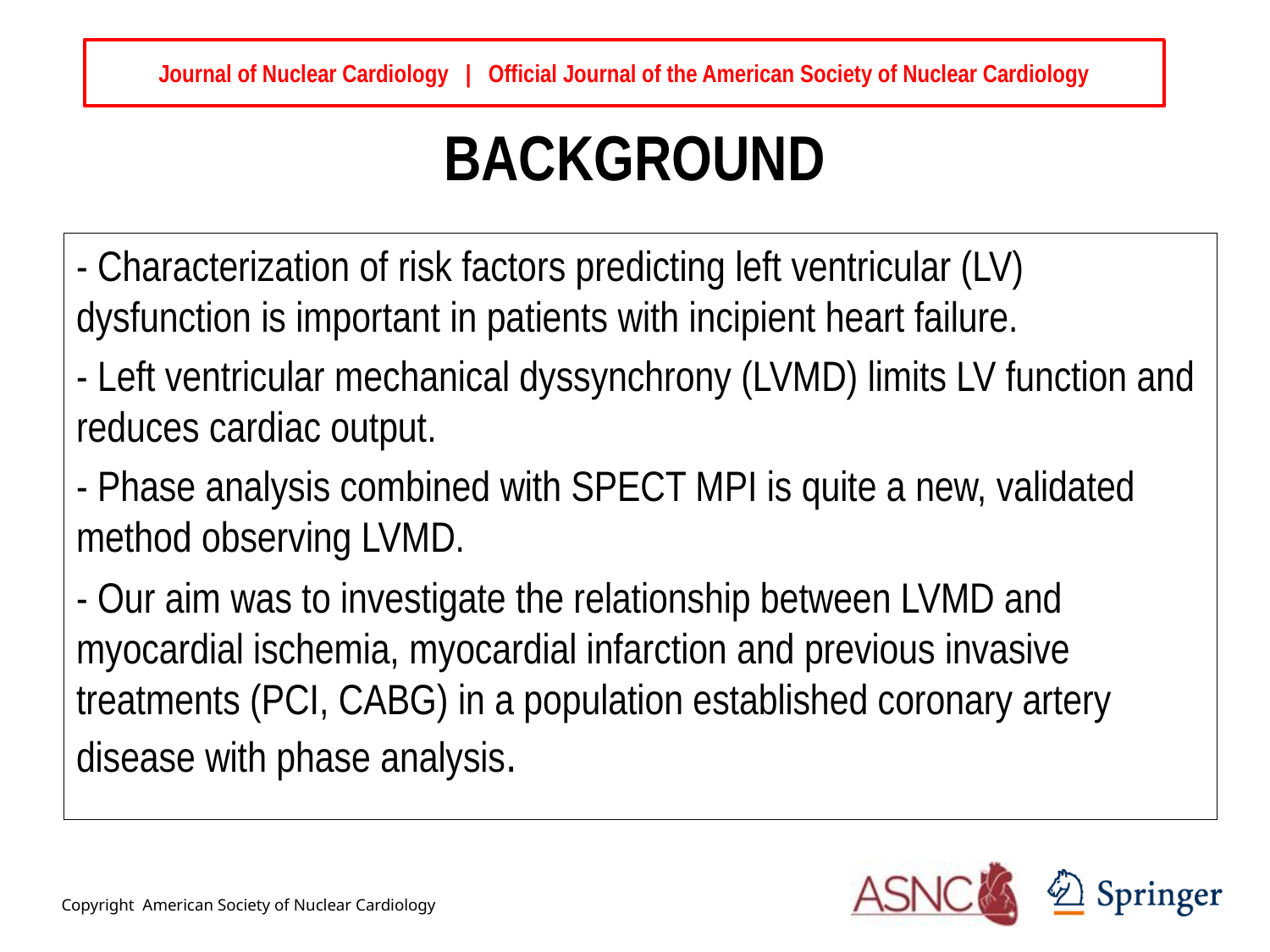

Journal of Nuclear Cardiology | Official Journal of the American Society of Nuclear Cardiology
# BACKGROUND
- Characterization of risk factors predicting left ventricular (LV) dysfunction is important in patients with incipient heart failure.
- Left ventricular mechanical dyssynchrony (LVMD) limits LV function and reduces cardiac output.
- Phase analysis combined with SPECT MPI is quite a new, validated method observing LVMD.
- Our aim was to investigate the relationship between LVMD and myocardial ischemia, myocardial infarction and previous invasive treatments (PCI, CABG) in a population established coronary artery disease with phase analysis.
Copyright American Society of Nuclear Cardiology

## Slide 3
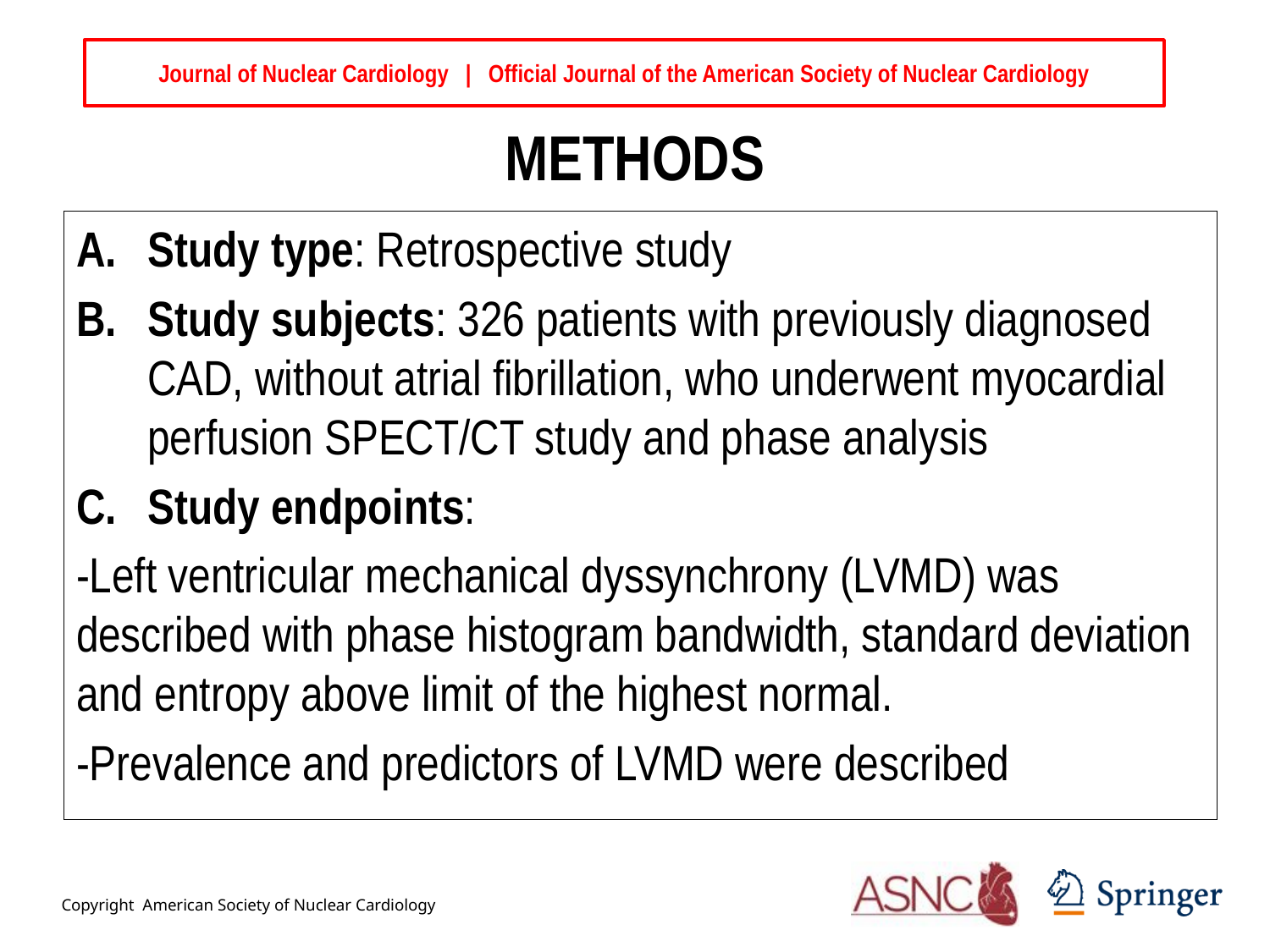

Journal of Nuclear Cardiology | Official Journal of the American Society of Nuclear Cardiology
# METHODS
Study type: Retrospective study
Study subjects: 326 patients with previously diagnosed CAD, without atrial fibrillation, who underwent myocardial perfusion SPECT/CT study and phase analysis
Study endpoints:
-Left ventricular mechanical dyssynchrony (LVMD) was described with phase histogram bandwidth, standard deviation and entropy above limit of the highest normal.
-Prevalence and predictors of LVMD were described
Copyright American Society of Nuclear Cardiology

## Slide 4
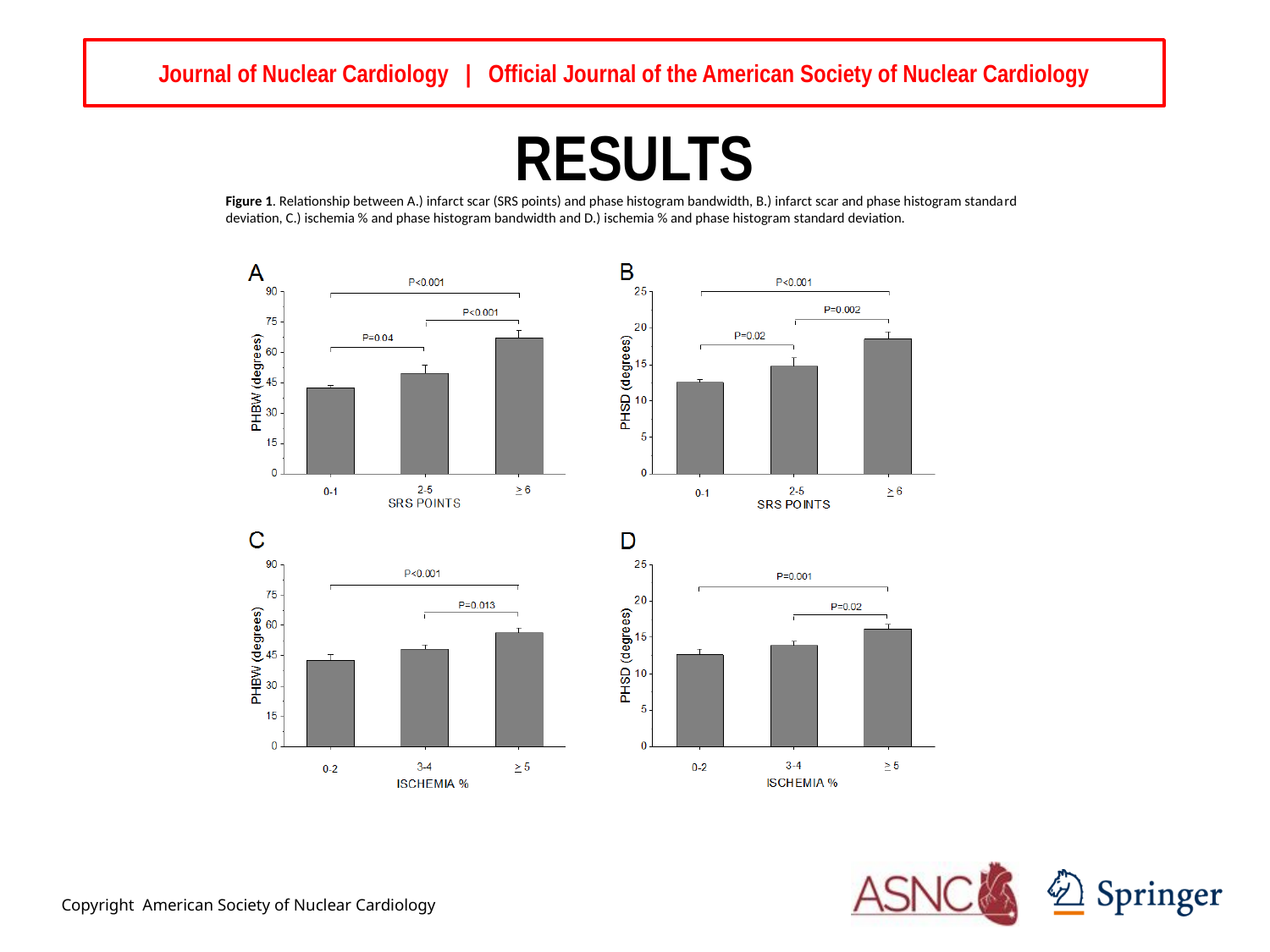

Journal of Nuclear Cardiology | Official Journal of the American Society of Nuclear Cardiology
# RESULTS
Copyright American Society of Nuclear Cardiology

## Slide 5
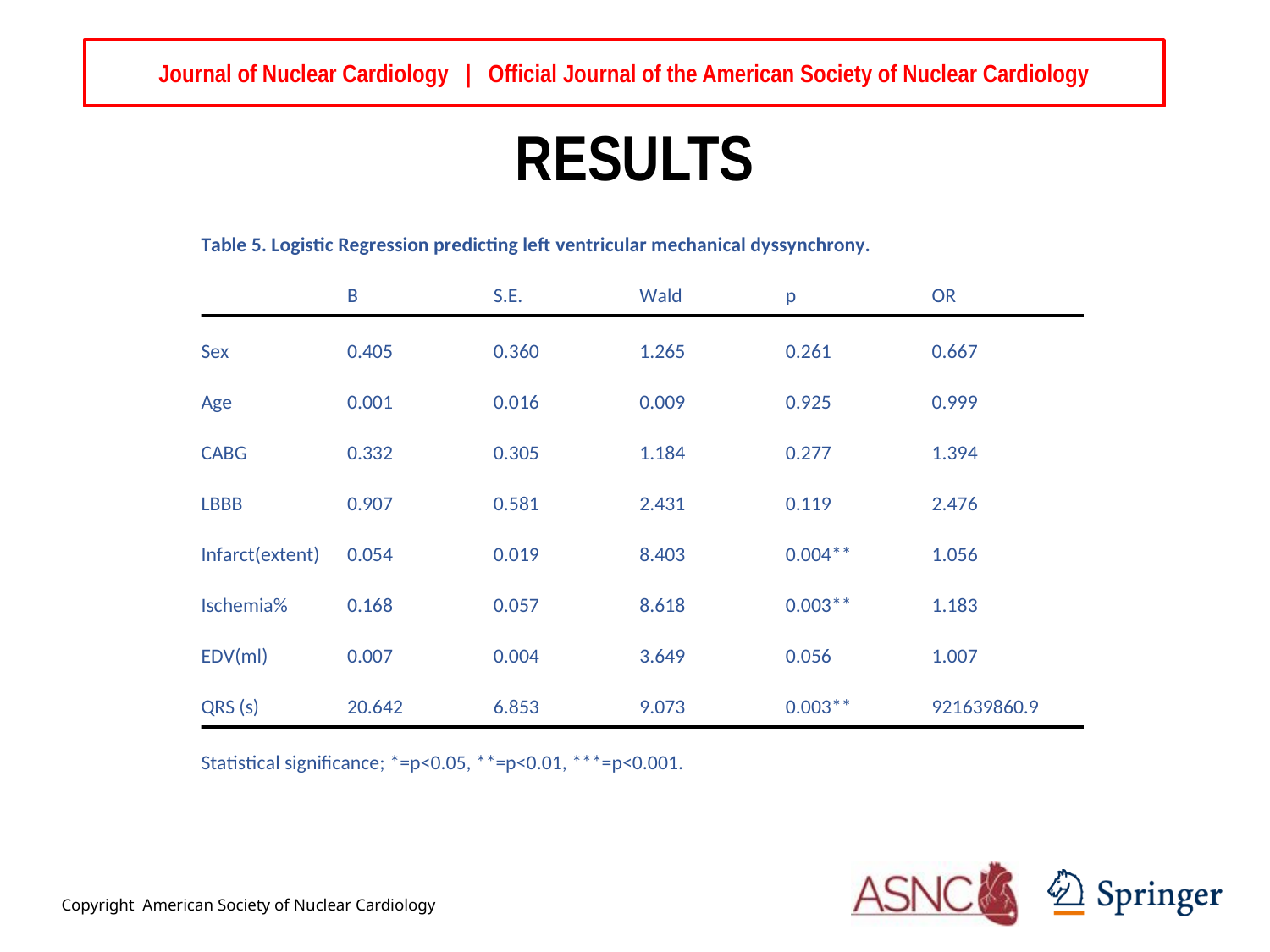

Journal of Nuclear Cardiology | Official Journal of the American Society of Nuclear Cardiology
# RESULTS
Copyright American Society of Nuclear Cardiology

## Slide 6
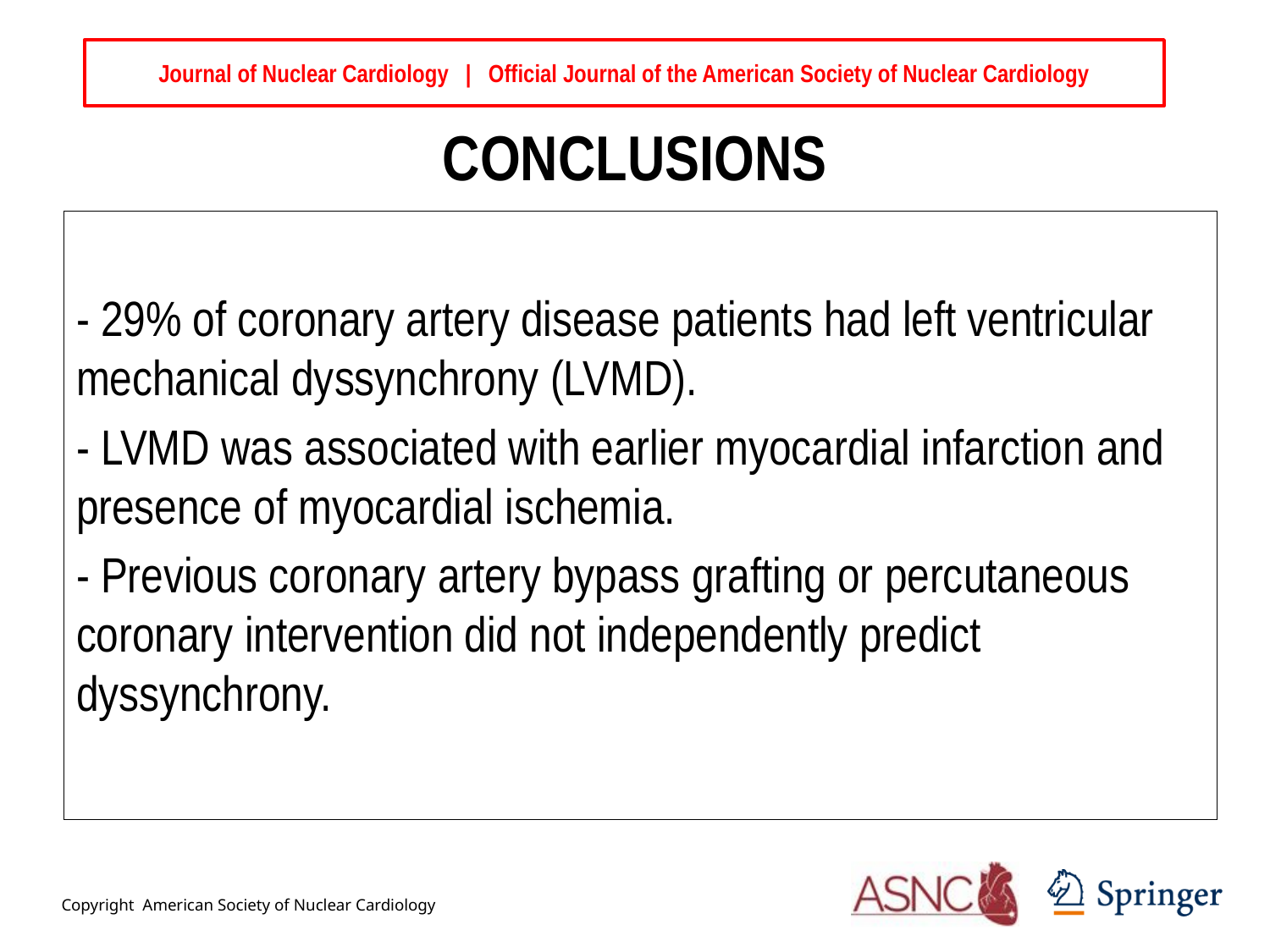

Journal of Nuclear Cardiology | Official Journal of the American Society of Nuclear Cardiology
# CONCLUSIONS
- 29% of coronary artery disease patients had left ventricular mechanical dyssynchrony (LVMD).
- LVMD was associated with earlier myocardial infarction and presence of myocardial ischemia.
- Previous coronary artery bypass grafting or percutaneous coronary intervention did not independently predict dyssynchrony.
Copyright American Society of Nuclear Cardiology
